# RNA structure mediated thermoregulation: What can we learn from plants?

**DOI:** 10.3389/fpls.2022.938570

**Published:** 2022-08-17

**Authors:** Sherine E. Thomas, Martin Balcerowicz, Betty Y.-W. Chung

**Affiliations:** ^1^Department of Pathology, University of Cambridge, Cambridge, United Kingdom; ^2^Division of Plant Sciences, The James Hutton Institute, University of Dundee, Dundee, United Kingdom

**Keywords:** RNA structure, plants, translation, protein synthesis, thermosensor, temperature

## Abstract

RNA molecules have the capacity to form a multitude of distinct secondary and tertiary structures, but only the most energetically favorable conformations are adopted at any given time. Formation of such structures strongly depends on the environment and consequently, these structures are highly dynamic and may refold as their surroundings change. Temperature is one of the most direct physical parameters that influence RNA structure dynamics, and in turn, thermosensitive RNA structures can be harnessed by a cell to perceive and respond to its temperature environment. Indeed, many thermosensitive RNA structures with biological function have been identified in prokaryotic organisms, but for a long time such structures remained elusive in eukaryotes. Recent discoveries, however, reveal that thermosensitive RNA structures are also found in plants, where they affect RNA stability, pre-mRNA splicing and translation efficiency in a temperature-dependent manner. In this minireview, we provide a short overview of thermosensitive RNA structures in prokaryotes and eukaryotes, highlight recent advances made in identifying such structures in plants and discuss their similarities and differences to established prokaryotic RNA thermosensors.

## Introduction

RNA molecules play vital roles in all extant forms of life: they serve as blueprints for protein synthesis, exert regulatory effects on DNA, other RNAs and proteins and even act as biocatalysts. Underpinning all these functions is their secondary and tertiary structure: inter- and intramolecular base pairing results in the formation of hairpins, apical and internal loops, three-way junctions and pseudo-knots (Cruz and Westhof, 2009). The critical role of these structures in non-coding RNAs has long been established, but over the years, it has become clear that they play important roles in transcription, processing, splicing, transport, translation as well as stability of mRNA (Jacobs et al., 2012; Lazzaretti and Bono, 2017; Soemedi et al., 2017; Bartys et al., 2019).

Thermodynamic free energy is a strong determinant of RNA secondary structure and thus, only the most energetically favorable will be adopted at any given time from a multitude of possible structures. While RNA folding is an intrinsic property of each RNA molecule, it is highly sensitive to the molecular environment and is influenced by RNA-binding proteins and ligands in addition to physical and chemical parameters. The free energy of RNA folding is directly dependent on temperature and therefore temperature changes can have profound and immediate consequences on RNA conformation. Transcriptome-wide RNA structure probing indeed revealed that shifts to higher temperature globally reduce RNA structure formation in several bacterial species (Righetti et al., 2016) as well as in yeast (Rouskin et al., 2014) and plants (Su et al., 2018).

Temperature affects every physical and biochemical process within the cell; it is thus vital for every organism to tightly monitor its temperature environment. Many organisms are known to undergo substantial physiological and developmental changes when their ambient temperature deviates from the desired optimum. As its structure is intrinsically thermosensitive, RNA represents an attractive candidate molecule to perceive temperature signals. While many conformational changes triggered by a rise or drop in temperature are likely to be subtle with limited functional consequences, some RNA structures undergo substantial rearrangements across a physiologically relevant temperature gradient, and it is these structures that can be harnessed by cells to monitor their temperature surroundings. Such RNA-based thermosensors are best described in bacteria, including plant symbionts and pathogens, where the so-called RNA thermometers control translation initiation in direct response to the temperature environment (Narberhaus et al., 2006).

However, thermosensory RNA elements in eukaryotes are not extensively explored to date. Previous studies on ectotherms such as *Drosophila* and *Trypanosomes* predict the possibility of mRNA 5′-UTR secondary structure mediated regulation in heat shock translation response (Ahmed and Duncan, 2004; David et al., 2010). Recent discoveries have shown that thermosensitive RNA structures that control gene expression *in cis* also exist in plants, although their mode of action differs from bacterial RNA thermometers (Chung et al., 2020). Temperate plants are exposed to a wide range of temperatures throughout the year and substantially change their growth habit and cellular composition in response to temperature changes. In the model plant *Arabidopsis thaliana*, warm ambient temperatures in the range of 25–30°C trigger increased elongation growth of stems and roots, reduced stomata formation and accelerated flowering (Nomoto et al., 2013; Song et al., 2018). These developmental adjustments largely aim at avoiding future heat and drought stress, but come at the cost of reduced immunity, particularly reduction in effector-triggered immune responses, at higher temperatures (Cheng et al., 2013; Menna et al., 2015; Huot et al., 2017; Qiu et al., 2022). Under severe heat stress above 40°C, growth instead ceases, and plants alter their physiology to minimize damage to membranes and proteins (Hayes et al., 2021). While these processes have been investigated for decades, studies have mainly focused on transcriptional or post-transcriptional mechanisms operating *in trans*, such as those mediated by miRNA, and on post-translational control, little attention has been paid to direct regulation of translation *in cis* by mRNAs (Megha et al., 2018).

Recently however, a genome-wide analysis of rice (*Oryza sativa* L.) seedlings reveals global structural changes in the transcriptome in response to high temperature due to unfolding of mRNA 5′ and 3′-untranslated regions (UTRs) (Su et al., 2018). This results in reduced mRNA stability and downregulation of global translation in rice seedlings. Temperature dependent splicing can be mediated by alterations of pre-mRNA secondary structures at intron-exon junctions thereby modulating the activity of spliceosome complex or that of splicing regulators (Shiina and Shimizu, 2020). Several examples of this mechanism are being identified, including alternative splicing of pre-mRNA encoding for an amino peptidase in *Saccharomyces cerevisiae* (Meyer et al., 2011) and more recently at the intron 2 of Heat shock factor (HsfA2) pre-mRNA from *Solanum lycopersicum* (Broft et al., 2022). Yet another study shows that thermosensitive RNA structures directly influence protein synthesis *in cis* in *Arabidopsis*, highlighting the importance of translational control in plant temperature responses (Chung et al., 2020). Likewise, increased RNA G-quadruplex (RG4) formation in 3′-UTRs of plant transcripts, in response to low temperatures, have been shown to enhance mRNA stability and adaptation to cold environment (Yang et al., 2022).

In this minireview, we compare recently identified thermosensitive RNA structures in plants with structures found in prokaryotic and other eukaryotic systems and contrast the different modes of action employed by these structures to control gene expression.

## Prokaryotic RNA thermometers

**FIGURE 1:**
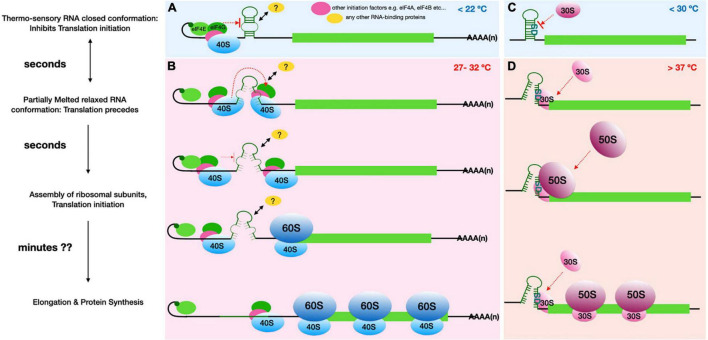
Comparison of the proposed plant RNA ThermoSwitch mechanism **(A,B)** with that of known Prokaryotic thermometers **(C,D)**. **(A)** The plant RNA ThermoSwitch adopts a closed conformation at temperatures below 22°C, at which cap-dependent recruitment of 43S complex occurs, however further scanning and identification of the start codon is impeded by the ThermoSwitch. **(B)** When temperature reaches between 27 and 32°C, the ThermoSwitch RNA undergoes reversible partial melting, thereby adopting a relaxed conformation and allowing the 43S complex to precede scanning. The partially open conformation temporarily impedes further incoming scanning subunits until the preceding 43S complex encounters the start codon with sufficient initiation context, thereby facilitating 48S initiation complex formation followed by recruitment of the 60S ribosomal subunit. Thus, the switch from closed (<22°C) to relaxed conformation at 27-32°C may enhance translation whereby translation initiation rates are selectively increased at higher temperatures (Chung et al., 2020). The fully mature 80S ribosomes then enter the elongation stage of translation allowing protein synthesis, by which time (∼minutes) the ThermoSwitch RNA fully melts accompanied by the helicase activity of incoming scanning 43S complexes. It is also possible that the ThermoSwitch recruits an RNA binding protein, either at the stable conformation to further stabilise the ThermoSwitch at lower temperature (<22°C), or at the relaxed conformation to enhance 43S scanning. **(C)** The prokaryotic RNA thermometers operate by a distinct mechanism, since translation initiation here is dependent on the direct interaction of 16S rRNA within the 30S ribosomal subunit and the SD-sequence. The bacterial RNA thermosensors are typically located very close to the start codon and encompass the SD sequence preventing access to the initiating small ribosomal subunit. **(D)** At higher temperatures (>37°C), the RNA secondary structure either melts (e.g., ROSE element) or switches into an alternative structure (e.g., CspA cold shock transcript); both processes relieve the SD sequence from intra-molecular base pairing to allow interactions with the 30S ribosomal subunit and recruitment of 50S ribosomal subunit (Kortmann and Narberhaus, 2012). The fully assembled 70S ribosomes then proceed to elongation. Thus, translational induction mediated by prokaryotic RNA thermometers in cis is generally proportional to temperature: the higher the temperature, the less stable is the respective RNA secondary structure and therefore the more efficient is translation initiation. In both instances, translation initiation occurs within seconds of temperature rise while entry into elongation and complete protein synthesis may occur within minutes.

Temperature-dependent changes in RNA secondary and tertiary structures are effectively utilized by many bacteria to modulate gene expression. These RNA thermometers form a powerful tool for rapid control of bacterial protein synthesis in response to cellular events such as entry into host, heat or cold stress (Narberhaus et al., 2006). Bacterial thermoregulatory RNAs are typically found at the 5′-UTR (5′-UTR) of the corresponding mRNA and encompass the Shine-Dalgarno (SD) sequence. As depicted in Figure 1, upon temperature dependent melting of these secondary structures, these non-coding mRNA regions are unmasked, providing accessibility for the 30S ribosomal subunits to interact with the SD sequence and identify the start codon, thereby resulting in translation initiation (Kortmann and Narberhaus, 2012; Somero, 2018).

A well-studied class of bacterial RNA thermometers are the ROSE (repression of heat shock gene expression) elements which control the expression of small heat shock genes. Commonly found in Proteobacteria, these 5′-UTR hairpins undergo gradual melting in response to increasing temperature, which then leads to complete melting at high temperature (∼42°C) and full liberation of the SD and AUG start codon, allowing enhanced translation (Chowdhury et al., 2006; Waldminghaus et al., 2009). These mRNA regions often consist of up to 4 hairpins of varying stability. For example, the *Escherichia coli ibpA* RNA thermometer, which regulates expression of an inclusion-body associated protein, encompasses three RNA hairpins at the 5′-UTR. The two proximal hairpins were found to be stable at temperatures ranging from 20 to 50°C and may be involved in proper folding of the distal temperature sensitive hairpin encompassing the SD sequence and start codon (Waldminghaus et al., 2009).

### RNA thermometers in prokaryotes: Plant pathogens and symbionts

RNA-based thermometers are effectively utilized by several plant pathogens and symbionts to regulate gene expression. Particularly well-studied in this context is the Rhizobiaceae family of plant symbiotic bacteria. In many rhizobial species, the expression of heat-shock genes is under the control of *cis*-acting ROSE elements. Structural and mutational studies on the small heat shock protein (HspA) transcript from *Bradyrhizobium japonicum*, a nitrogen-fixing root nodule bacterium, revealed a functional ROSE domain in the 5′-UTR that encompasses the transcript’s SD sequence. This domain forms an elaborate secondary structure consisting of four hairpins, which help in the ROSE at temperatures <30°C (Nocker et al., 2001a). Upon heat shock (∼42°C), the SD sequence gets exposed, allowing binding of the ribosomal 30S subunit.

Several non-canonical base pairs and transient hydrogen bonds are observed in all known ROSE-type thermometers in addition to a conserved short stretch of nucleotides UYGCU near the SD sequence. These secondary mRNA structure features are also observed in the heat-sensitive RNA structures of many other rhizobia such as *Bradyrhizobium* sp. *(Parasponia), Mesorhizobium loti*, and are functionally interchangeable among the species (Nocker et al., 2001a,b). High resolution NMR structures of the *B. japonicum hspA* 5′-UTR shows that the corresponding G83 nucleotide is involved in non-canonical base *syn–anti* base pairing with G94 in the SD region. In fact, the entire stretch of nucleotides complementary to the SD sequence are conserved in all known ROSE-like RNA elements in *rhizobia* (Chowdhury et al., 2006).

Similarly, translation of pAT-plasmid encoded small heat shock genes in *Agrobacterium tumefaciens*, plant pathogenic bacteria, is also mediated by ROSE-controlled expression. The heat shock gene transcripts *hspAT1* and *hspAT2* exhibit the characteristic features of ROSE-thermometers such as masked SD sequences and hairpin base pairing. In addition, the strictly conserved bulged G nucleotide opposite the SD sequence is observed in computer-aided secondary structure predictions of *hspAT1* and *hspAT2* transcripts (Balsiger et al., 2004). Thus, these RNA thermometers have evolved a conserved mechanism that is sufficient to control rapid synthesis of several heat-shock proteins in response to temperature.

### Switch-like thermoregulatory RNA in the prokaryotic cold shock response

In addition to the zipper-like RNA thermometers discussed so far, many bacteria contain a more switch-like thermoregulatory mechanism involving two mutually exclusive conformations. Examples of these are seen in bacterial cold shock responses such as in the expression of the CspA RNA chaperone in *E. coli.* The *cspA* transcript is unstable at 37°C but is highly stable when temperature drops to 10°C owing to reorganization of the mRNA thermosensory elements located at the 5′-UTR and extending a further 60 nucleotides downstream into the coding region. The rearrangements result in an alternate, mutually exclusive *cspA* mRNA conformation in response to cold shock, which in turn is efficiently translated due to the now highly exposed translation initiation sequence elements and low susceptibility to RNA degradation (Giuliodori et al., 2010; Phadtare and Severinov, 2010; Kortmann and Narberhaus, 2012).

### RNA thermometers in prokaryotic virulence transcripts

Other well-characterized examples of bacterial RNA thermometers include those controlling virulence factor genes such as the mRNAs encoding *Listeria monocytogenes* virulence regulator PrfA (Johansson et al., 2002) and *Yersinia* virulence factor LcrF (Skurnik and Toivanen, 1992; Hoe and Goguen, 1993; Bohme et al., 2012; Pienkoss et al., 2021). In these pathogenic bacteria, a rapid temperature shift from low temperatures (<30°C) to 37°C, experienced upon host entry, triggers expression of the respective virulence factor genes. These protein factors in turn help evade immune responses and establish successful infection in the host. The 5′-UTR of *Yersinia pestis lcrF* controls access to the SD sequence *via* a small zipper-like RNA hairpin consisting of four consecutive uridines which base-pair with the SD sequence. Together with the *Salmonella enterica* small heat shock gene *agsA* mRNA 5′-UTR thermometer, this represents one of the founding members of the *fourU* type thermoregulatory RNA elements (Hoe and Goguen, 1993; Waldminghaus et al., 2007).

Interestingly, the *L. monocytogenes prfA* introduced above encompasses an elaborate zipper mechanism, which, in addition to encoding an elaborate temperature-controlled RNA secondary element in its 5′ leader, couples a truncated SAM riboswitch element (SreA), a *trans*-acting small RNA (sRNA). This sRNA in turn responds to elevated levels of S-adenosyl methionine (SAM) such as when inside the mammalian host, and functions *in trans* by binding to the *prfA* RNA thermosensor causing repression of PrfA synthesis. Thus, an additional feedback signal is integrated to regulate translation of the virulence regulator PrfA, thereby fine-tuning expression of several virulence genes and host immune modulators (Hoe and Goguen, 1993; Johansson et al., 2002; Bohme et al., 2012; Kortmann and Narberhaus, 2012).

## RNA structure-mediated thermosensing in eukaryotes

In contrast to bacterial RNA thermometers, similar mechanisms in *eukarya* and *archaea* are only beginning to be understood. Some of the known or predicted eukaryotic thermoregulatory RNA mediates translation in response to temperature fluctuation, whether within physiological range, heat stress or cold shock. Rapid increase in temperature often leads to toxic accumulation of misfolded proteins which, if unchecked, can severely compromise cellular function even leading to apoptosis (Lindquist, 1986). A characteristic response to elevated temperatures involves the repression of normal cell metabolic processes and rapid production of large amounts of stress proteins known as heat shock proteins (HSPs) (Lindquist, 1981, 1986; Ahmed and Duncan, 2004).

The possibility of a rapid-heat inducible RNA structural element was predicted in the *Hsp90* transcripts in *Drosophila* (Ahmed and Duncan, 2004). Interestingly, the *Drosophila Hsp90* mRNA 5′-UTR consists of extensive secondary structure regions in contrast to other HSP mRNAs (*Hsp70* and *Hsp22*) (Ahmed and Duncan, 2004). Despite the importance of these rapid stress responders in cellular homeostasis, the detailed mechanism of the *Hsp90* mRNA structure mediated regulation of this heat shock response in *Drosophila* and other eukaryotes is poorly understood.

### Heat-shock RNA1: A *trans*-acting structured RNA thermosensor in eukaryotes

The upregulation of HSPs plays a crucial role in ameliorating such lethal effects by assisting proper folding of damaged proteins, allowing repair, localization, and transport of proteins and re-establishing the equilibrium (Taipale et al., 2010). Under stress stimuli, the HSPs preferentially bind to aggregated misfolded proteins releasing HSFs (heat shock factors), allowing their translocation to the nucleus and trimerization into the active form, in turn inducing expression of HSPs. Once the stress induced protein damage to the cells is relieved, the HSPs negatively regulate their own synthesis by interacting with HSFs (Solis et al., 2016; Schopf et al., 2017). Recent studies have identified a small *trans*-acting RNA forming an essential regulatory component of the mammalian heat-shock-factor-1 (HSF-1) by assisting autoregulation of HSPs. This RNA element, termed heat-shock RNA1 (HSR1), undergoes temperature-dependent changes in conformation driving activation of HSF-1 leading to transcription of heat-shock protein genes. In addition to humans and rodents, these highly conserved HSR1 sequences were also identified in *Drosophila*, *Caenorhabditis elegans*, *Arabidopsis*, and bacteria (Shamovsky et al., 2006; Choi et al., 2015; Somero, 2018).

### 3′-UTR RNA secondary structures in eukaryotic thermosensing

Eukaryotic heat shock responses can be affected by temperature-sensitive RNA structures, often found in the 3′-UTR of the corresponding transcripts. Examples of these type of control have been reported in *Leishmania* and rice *(O. sativa* L.), where they modulate RNA stability and consequently affect translation rates of the associated coding region.

A recent genome-wide study of heat shock response in rice shows that RNA secondary structure reprogramming broadly regulates protein synthesis at elevated temperatures (Su et al., 2018). In this study a dimethyl sulfate (DMS) based structure-seq analysis of rice (*O. sativa* L.) seedlings revealed global structural changes in the transcriptome at elevated temperature of 42°C due to RNA unfolding. However, a parallel Ribo-seq analysis showed no correlation between RNA unfolding and heat-induced changes in translation, while RNA-seq based transcript quantification indicates that the observed structural changes promote transcript degradation. Hence the authors conclude that the downregulation of global translation in rice, which reduces heat-stress induced damage, is facilitated by increased access of the RNA degradation machinery to unfolded mRNA 5′ and 3′-UTRs. Yet another study analyzed the nucleotide compositions of transcriptomes of 906 land plants (Yang et al., 2022) and found that plants growing in cold climates have guanine (G)-enriched transcripts. These transcripts in turn have high propensity to form RG4 structures. Further *in vivo* structure probing in *Arabidopsis* combined with immunofluorescence studies show that the RG4 formation in 3′-UTRs of plant transcripts, which are globally increased in response to low temperature (∼ 4°C), lead to enhanced mRNA stability and adaptation to cold (Yang et al., 2022).

Studies on *Leishmania* heat shock protein *Hsp83* transcripts identified nucleotides 1–472 in the proximal 3′-UTR as having thermoregulatory function (Larreta et al., 2004; David et al., 2010). Translation regulation is effectively utilized by digenetic parasites like *Leishmania* for differential expression of genes during their life cycle. These parasites undergo differentiation from promastigote stage, in the alimentary tract of sandfly, to amastigotes once phagocytosed by macrophages of the mammalian host (Larreta et al., 2004). Adaptation to these distinct environments is achieved largely by controlling post-transcriptional events such as mRNA processing, stability and through translation regulation. Preferential translation of trypanosomatids in response to environmental cues such as temperature and pH, are heavily mediated by 3′-UTR of stage-specific transcripts (Zilka et al., 2001).

The regulatory element within this proximal 3′-UTR region consists of a long polypyrimidine tract (PPT) located between positions 312 and 341. Computer-based structure predictions showed that this region is positioned on a highly probable secondary structure. Thermal melting profiles at 260 nm and RNAase H assays of WT and mutated 3′-UTRs suggest that this regulatory region undergoes partial melting during a temperature shift from 26 to 37°C, indicating the presence of a thermosensory RNA sequence. The study also shows that *Hsp83* preferential translation requires scanning of the *Hsp83* 5′-UTR, unlike cap-independent translation of Hsps of many higher eukaryotes at elevated temperatures (Hernandez et al., 2004; Duncan, 2008; David et al., 2010). While a detailed study of the molecular mechanism of this thermoregulatory PPT in the *Leishmania Hsp83* transcript 3′-UTR is pending, it is hypothesized that the temperature-mediated exposure of this region results in binding of regulatory factors such as PPT binding protein (PTB), allowing interactions with the translation initiation complex. This may in turn lead to circularization of the mRNA by bridging of the 5′ and 3′-ends, thereby enhancing protein synthesis at elevated temperatures (Gingras et al., 1999; David et al., 2010; Mishra et al., 2020).

## Translational control through plant RNA ThermoSwitches

Plants have evolved various strategies for adaption to changes in surrounding temperatures. A recent study in the model plant *Arabidopsis* demonstrates, for the first time, the presence of plant RNA ThermoSwitches which directly control the expression of several transcripts in response to warm temperatures (Chung et al., 2020). This study thus illuminates the existence of an mRNA structure-mediated control mechanism in response to temperature changes during eukaryotic translation. The temperature-dependent translational enhancement in *Arabidopsis* has been investigated in detail for transcript encoding transcription factor PIF7 (PHYTOCHROME INTERACTING FACTOR 7): it is mediated by the formation an mRNA hairpin in the 5′-UTR, approximately 30 nucleotides upstream of the start codon. Further biophysical studies combined with *in vitro* translation experiments suggest that the hairpin remains stable at temperatures <22°C but switches to a partially melted open conformation at temperatures of 27–32°C. This structural change results in a direct increase in PIF7 protein synthesis, and high PIF7 protein levels in turn promote the transcription of several downstream genes, including those of the auxin biosynthetic pathway, thereby enhancing elongation growth during warm daytime (Chung et al., 2020).

Similar hairpin sequences were identified in the 5′-UTRs of several other transcription factors such as WRKY22 and the heat shock regulator HSFA2, suggesting a conserved regulatory mechanism enabling plants to elicit rapid adaptive response at temperatures within physiological range (Chung et al., 2020). Computational analysis of these mRNA 5′-UTR regions shows high structural similarity consisting of a hairpin involving 28 nucleotides in the distal half of this region, despite the lack of sequence conservation.

These plant RNA ThermoSwitches appear to operate differently from bacterial RNA thermometers or from the mutually exclusive thermo-switch conformations seen in many bacterial cold shock protein transcripts (Breaker, 2010; Kortmann and Narberhaus, 2012), as depicted in Figure 1. In the bacterial system, translation initiation is dependent on the direct interaction of the 16S rRNA within the 30S ribosomal subunit and the SD-sequence, a purine-rich sequence approximately 5 nt upstream of the start codon on the messenger RNA (Steitz, 1969; Shine and Dalgarno, 1974; Steitz and Jakes, 1975). With the help of prokaryotic initiation factors IF1, IF2, and IF3, this interaction leads to the recruitment of the initiator met-tRNA to the start codon, followed by recruitment of the 50S ribosomal subunit to assemble the initiating 70S ribosome, placing the start codon:met-tRNA in the P-site of the 70S ribosome (Rodnina, 2018). The bacterial RNA thermometers are typically located very close to the start codon and encompass the SD sequence. At high temperature, the RNA secondary structure either melts (e.g., ROSE element) or switches into an alternative structure (e.g., CspA cold shock transcript); both processes relieve the SD sequence from intra-molecular base pairing to allow interactions with the 16S rRNA on the 30S ribosomal subunit (Kortmann and Narberhaus, 2012). Thus, translational induction mediated by these RNA thermometers *in cis* is generally proportional to temperature: the higher the temperature, the less stable is the respective RNA secondary structure and therefore the more efficient is translation initiation (Figure 1).

In contrast, hallmarks of canonical eukaryotic translation initiation involve scanning of the 43S complex through the 5′-UTR (Kozak, 1989; Jackson et al., 2010). Eukaryotic translation initiation is a highly involved multi-step process starting with recognition of the 5′ cap on the mRNA by the eIF4F complex, which leads to the recruitment of the 43S complex, comprising the 40S subunit and several initiating factors, including the eIF2-GTP-Met-tRNA*^Met^*i complex, to the 5′ end of the mRNA to begin the scanning process. Scanning by the 43S complex consists of two linked processes: movement of the 43S complex in a strictly 5′ to 3′ dependent manner and unwinding of secondary structures at the same time. Initiation then begins once the 43S complex encounters a start codon with sufficient initiation context, particularly with a purine at −3 and +4 position also known as Kozak consensus. The process is facilitated by eukaryotic initiation factors eIF1 and eIF1a and places the eIF2-GTP-Met-tRNA*^Met^*i with the start codon through base pairing with the tRNA anticodon followed by joining of the 60S ribosomal subunit. At higher temperatures the incoming scanning 43S complex is temporarily impeded by the relaxed, partially melted RNA ThermoSwitch, instead of interrupting the process of 48S Initiation complex formation facilitating recruitment of 60S ribosomal unit and assembly of the fully mature 80S ribosome to enter the elongation stage of translation (i.e., protein synthesis). Therefore, rather than providing accessibility for the ribosomal subunits to join the mRNA, the plant RNA ThermoSwitch is likely to regulate protein synthesis through the scanning process, possibly assuming a dual role: while its stable structure at temperatures less than 22°C may well impede movement of the scanning complex, its more relaxed structure at 27–32°C acts as a translational enhancer, increasing translation initiation rates selectively at these temperatures (Figure 1). This mode of action is evidenced by the fact that both disrupting and stabilizing the hairpin structure has a detrimental effect on translation rates and protein synthesis (Chung et al., 2020). The precise mechanism by which the ThermoSwitch enhances translation remains to be determined. RNA secondary structures are known to regulate RNA–RNA (Van Treeck and Parker, 2018) and RNA-protein interactions (Sanchez De Groot et al., 2019); such interactors may include RNAs or RNA-binding proteins that directly facilitate translation initiation, or proteins that affect RNA modification or localization and thereby indirectly influence the initiation process. In case of both prokaryotic and known eukaryotic thermoregulatory RNAs, the temperature mediated conformational change in RNA secondary structure and translation initiation happens within seconds of temperature rise while entry into elongation and complete protein synthesis may occur within minutes. Table 1 summarizes the known and predicted RNA structure mediated thermo-sensors discussed in this review.

**TABLE 1:** Summary of known and predicted RNA structure mediated thermo-sensors discussed in this review.

Thermosensor	Organism	Function	Mechanism	Temperature range	References
**Prokaryotic**					
*prfA*	*Listeria monocytogenes*	Bacterial virulence factor, helps evade immune repose	Zipper-like 5′-UTR RNA hairpins consisting of four consecutive uridines (*fourU* elements) controlling access to SD sequence	<30°C to 37°C experienced during mammalian host entry	Johansson et al., 2002
*lcrF*	*Yersinia pestis*	Bacterial virulence factor for establishing successful infection	5′-UTR thermosensory RNA Zipper coupled to *trans*-acting small RNA (SAM riboswitch element; SreA) triggered by elevated levels of SAM found in the human host	<30°C to 37°C experienced during mammalian host entry	Hoe and Goguen, 1993
*agsA*	*Salmonella*	Heat shock response	*FourU* 5′-UTR RNA zipper. Temperature-dependent melting also modulated by hydration shell and ion concentrations		Waldminghaus et al., 2007
*ibpA*	*Escherichia coli*	Heat shock response	ROSE (repression of heat shock gene expression) 5′-UTR RNA which controls expression of small heat shock genes	Gradual melting at heat shock temperatures. Complete melting and full liberation of the SD sequence and start codon at ∼42°C	Waldminghaus et al., 2009
*cspA*	*Escherichia coli*	Cold shock response	Switch-like thermoregulatory mechanism involving two mutually exclusive conformations		Giuliodori et al., 2010
*hspA*	*Rhizobiaceae*	Heat shock response	ROSE elements with 2–4 hairpins, functional ROSE domain, encompassing the SD sequence, located at hairpin in the distal end of the 5′-UTR	Repression at non-heat shock temperatures <30°C, gradual melting at higher temperatures, complete melting at ∼42°C	Nocker et al., 2001a; Chowdhury et al., 2006
*hspAT*	*Agrobacterium tumefaciens*	Heat shock response	Characteristic features of ROSE-controlled 5′-UTR RNA thermometers such as masked SD sequence and with a complementary conserved short stretch of UYGCU nucleotides.	Translation repression at <30°C, gradual melting at higher temperatures, complete melting at ∼42°C	Balsiger et al., 2004
**Eukaryotic**					
*PIF7, HSFA2, WRKY22*	*Arabidopsis thaliana*	Growth response to warm day cycles (PIF7), heat shock response (HSFA2), stress response (WRKY22)	mRNA 5′-UTR hairpin impeding scanning by 43S complexes at low temperatures. Translation is enhanced by switch of the hairpin from close to open conformation in response to warmer temperatures.	Low translation at temperatures <22°C, partial melting, and optimal translational response at 27–32°C	Chung et al., 2020
*Hsr1*	Human Mouse *Drosophila* *Caenorhabditis elegans*	Heat shock response	Heat-sensing non-coding RNA that undergoes temperature-dependent changes in secondary structure, driving activation of heat shock factor (HSF-1) and transcription of heat-shock protein genes	Heat shock temperatures 42–45°C	Shamovsky et al., 2006; Choi et al., 2015
*Hsp90 (predicted)*	*Drosophila melanogaster*	Heat shock response	Predicted mRNA 5′-UTR thermo-sensor consisting of extensive secondary structure regions	Enhancement of *Drosophila* Hsp90 translation observed at ∼30–37°C. Optimal expression at 35°C	Ahmed and Duncan, 2004
*Hsp83 (predicted)*	*Leishmania*	Heat shock response	Predicted 3′-UTR thermo-sensor consists of long polypyrimidine tract (PPT). Temperature-mediated exposure possibly allowing interaction with the 5′-UTR *via* transacting factors.	Partial melting of 3′-UTR observed with temperature shift from ∼26°C to 37°C	Larreta et al., 2004; David et al., 2010

## An exciting future for thermosensory plant RNA structures?

The recent discovery of thermosensory RNA structures in plants emphasizes the important regulatory role RNA can play in the control of gene expression. Notably, we now know that RNA structures as part of thermosensory signaling modules are not confined to prokaryotes, but may be prevalent in eukaryotic systems, opening a new chapter of research into thermosensor research. It will be exciting to see whether similar thermosensory structures will be discovered in other eukaryotic species and how such structures have been harnessed by different organisms to tailor their development and physiology to their surrounding temperatures.

In addition, thermosensory RNA structures may provide a new means to manipulate gene expression to improve plant fitness and crop yield. It might become possible to tailor a gene’s expression level to specific temperature regimes by the addition of 5′-UTR thermoswitches or 3′-UTR G-quadruplexes. Temperature regulation of these structures is quick and, in case of thermoswitches, reversible, allowing for precise fine-tuning of temperature responses. The required structures could be introduced through gene editing techniques, avoiding the need to generate transgenic plants.

Likewise, plant RNA thermosensors can be utilized for advanced synthetic biology applications such as in inducible expression of plant pigments, phytochemicals and other biologicals of interest to biotechnological, pharmaceutical and food industry. Some recent studies involving the development of novel inducible riboswitches for metabolic engineering in green alga *Chlamydomonas reinhardtii* (Mehrshahi et al., 2020) and in tobacco (*Nicotiana tabacum*) plastids (Agrawal et al., 2022) are encouraging advances toward this aim. Further understanding of the essential features of these molecular thermometers and their precise functional mechanisms will facilitate future development of such biotechnological toolkits.

## Author contributions

BC proposed the manuscript. BC and ST researched the content. All authors contributed to writing of the manuscript and approved the final version.
